# Results of a hypertension and diabetes treatment program in the slums of Nairobi: a retrospective cohort study

**DOI:** 10.1186/s12913-015-1167-7

**Published:** 2015-11-17

**Authors:** Marie E. Werner, Steven van de Vijver, Mildred Adhiambo, Thaddaeus Egondi, Samuel O. Oti, Catherine Kyobutungi

**Affiliations:** University of Amsterdam, Amsterdam, The Netherlands; African Population and Health Research Center, Nairobi, Kenya; Department of Global Health, Academic Medical Center, Amsterdam, The Netherlands, and Amsterdam Institute for Global Health and Development, Amsterdam, The Netherlands

**Keywords:** Hypertension, Diabetes, Treatment, Cardiovascular diseases, Community-based, Sub-Saharan Africa, Kenya, Slum

## Abstract

**Background:**

Cardiovascular diseases (CVD) are the world’s leading cause of death and their prevalence is rising. Diabetes and hypertension, major risk factors for CVD, are highly prevalent among the urban poor in Africa, but treatment options are often limited in such settings. This study reports on the results of an intervention for the treatment of diabetes and hypertension for adult residents of two slums in Nairobi, Kenya.

**Methods:**

After setting up two clinics in two slums in Nairobi, hypertension and/or diabetes patients were seen by a clinician monthly. Socio-demographic characteristics and clinical data were collected over a 34-month period. Records were analyzed for 726 patients who visited the clinics at least once to determine clinic attendance and compliance patterns using survival analysis. We also examined changes in systolic blood pressure (SBP), diastolic blood pressure (DBP) and random blood glucose (RBG) during the course of the program.

**Results:**

There was poor compliance with clinic attendance as only 3.4 % of patients attended the clinics on a regular (monthly) basis throughout the 34-month period. 75 % of hypertension patients were not compliant after four visits and 27 % of patients had only one clinic visit. Significant reduction of mean SBP and DBP (150.4 mmHg to 141.5 mmHg, *P* = .003, and 89.3 mmHg to 83.2 mmHg, *P* < .001) was seen for all patients that stayed in care for at least one year.

**Conclusions:**

Establishing a preventative care and treatment system in low resource settings for CVD is challenging due to high dropout rates and non-compliance. Innovative strategies are needed to ensure that benefits of treatment programs are sustained for long-term CVD risk reduction in poor urban populations.

## Background

Hypertension is the leading risk factor for CVD, and its prevalence is increasing worldwide, from over 25 % in 2000 to a projected 40 % in 2025 [1, 2]. In Kenya the mean systolic blood pressure has increased from 127 to 132 mmHg between 1990 and 2008 [3]. Although awareness on hypertension seems to be low among the Kenyan population and other developing countries [1, 4–6], recent findings in Nairobi suggest that when people are made aware of having hypertension, they tend to seek care for it [1]. Also, improved physical access to health facilities leads to higher use of them [7] and has been reported as a contributor in reducing the overall CVD risk [8]. An intervention program that improves awareness for CVD risk factors and increases access to healthcare might therefore reduce the burden of CVD.

For diabetes, it is estimated that the global prevalence will increase from 6.9 % in 2010 to 7.7 % in 2030 [9]. The majority of this increase will occur in LMICs and the urban poor population is likely to be most affected [10, 11]. A recent study among residents of two Nairobi slums showed an age-adjusted prevalence of 5.5 % but with significant age differences. For instance, in women over the age of 60 years, the prevalence was 15 % [12]. Another study carried out in a different slum in Nairobi showed similar prevalences, compared to a prevalence of 2.2 % in rural areas [13]. Among patients with diabetes in urban slum communities in Nairobi the odds for hypertension were two fold, resulting in 45.5 % of patients with diabetes being hypertensive. Similarly, 13 % of patients with hypertension in these communities also had diabetes [9].

Urban settings in general have a higher risk of developing CVD, largely due to adoption of sedentary lifestyles, dietary changes and psychosocial stress [14–17]. UN-HABITAT reports that approximately 58 % of the Kenyan urban population lived in slums or slum-like conditions in 2008, characterized by poor living conditions and psychosocial stress [18, 19]. Considering the above and the fact that improved glycemic control and lowering blood pressures to normal values, both achievable via the use of antidiabetic and antihypertensive medication and lifestyle changes, can prevent CVD in the future [20], the African Population and Health Research Center (APHRC) executed a project from March 2009 to May 2012. This project aimed to provide better access to high quality hypertension and diabetes care and management in two slum communities underserved by public health facilities. The aim of this paper is to investigate whether this project can lead to health benefits for people in low resource settings, specifically by mapping the compliance patterns exhibited by the patients and the effect of clinic attendance on CVD risk measures such as SBP, DBP and random blood glucose (RBG).

## Methods

### Study setting

The project operated in two outreach clinics in two slums –Korogocho and Viwandani– in Nairobi. These clinics had been established out of necessity during a previous cross-sectional CVD risk factor assessment survey in both slums also implemented by APHRC. At the time of implementing the survey, there were no clinics attending to patients with hypertension and or diabetes in either slum. Therefore the APHRC set up two clinics within existing primary health care facilities in both slums. Participants in the cross-sectional survey who were found to have elevated blood pressure or elevated glucose values, or participants currently on medication for diabetes or hypertension, were referred to the clinics. At the clinics, adults diagnosed with hypertension and diabetes could obtain treatment and advice on self-management techniques. Elevated blood glucose levels were defined as; RBG ≥11.0 mmol/L [21]. Hypertension was defined as; SBP ≥ 140 mmHg and/or DBP ≥ 90 mmHg or currently on anti-hypertensive medication [22]. The clinics also attended to walk-in patients who had not been referred from the cross-sectional survey but had elevated blood pressure or glucose levels as defined above and met the rest of the inclusion criteria.

The project provided the two clinics with basic diagnostic equipment (digital blood pressure monitors, glucometers) and qualified health personnel (clinical officers and nurses). The clinics were open on a fortnightly basis. Patients were expected to attend the clinics at least once every four weeks or sometimes bi-weekly depending on the severity of their blood pressure or blood glucose readings. Patients who reached normal values were also advised to visit the clinic every four weeks. Each visit patients were seen by a physician and had their blood pressure and glucose measurements reassessed. They received lifestyle counseling, which included dietary and exercise advice and explanations of the risk factors and complications of the diseases. In addition, they received medication based on simple management guidelines. For a period of two years, until March 2011, receiving care and treatment at the clinics was absolutely free. Thereafter, a user fee was introduced. The fees for consultation, tests and a four weeks’ supply of drugs ranged from about 2$ for patients with hypertension to 6$ for patients with diabetes requiring insulin.

### Study design

This was a facility-based follow up study of patients enrolled in the two clinics. At the first visit, data collected included socio-demographic characteristics, diagnosis, anthropometry, blood pressure and fasting blood glucose. Each patient received a date for a follow up visit. At each subsequent visit, clinical measurements of weight, blood pressure and random blood glucose were taken and treatment was evaluated. These clinical data were later captured in an Epi-Info database.

Blood pressure was measured with validated oscillometric automated digital BP devices (OMRON Digital Automatic BP Monitor) used by qualified nurses or physicians [23]. Three measurements were taken on the left arm with one minute intervals while patients were seated and silent. Averages of the second and third measurements were used for analysis. Treatment of hypertension was defined as receiving prescribed antihypertensive medication for high blood pressure. Blood glucose was measured using glucometers (Accu-Chek Active Blood Glucose Meter System). Treatment of diabetes was defined as receiving prescribed insulin or oral hypoglycemic medication.

Since patients were typically given appointments four weeks after any visit, we defined compliance as attending the clinic within four weeks after the patient’s last visit, with patients being non-compliant when the timespan between two visits exceeded four weeks. We defined drop out of patients as failure to attend consecutive scheduled consultations from any point in time until the end of the 34-month period.

### Statistical analysis

The study participants were divided in three groups based on diagnosis: *hypertension patients*, *diabetes* patients and patients having *both hypertension and diabetes*. Descriptive analyses were conducted and group means were compared using unpaired two-tailed t tests, variance analysis (ANOVA) and chi-square tests. We looked at group differences in area of residence and sex. Bivariate analyses were conducted for the change in blood pressure and fasting blood glucose over time (6 and 12 months) using paired two tailed t tests, for patients who were enrolled in the project for these periods. Due to high levels of attrition, it was not possible to conduct any meaningful analyses beyond 12 months of follow up.

Power analyses were conducted to see whether the tests performed have an adequate power to detect statistical significance. Survival analysis was performed to evaluate when non-compliance in different groups occurred, with time to non-compliance being the event analyzed. In all analyses *P* < .05 was considered statistically significant. All analyses were performed using STATA 13.0 (Stata corp.).

### Ethical considerations

This study was approved by the Kenya Medical Research Institute/National Ethical Review Committee (NON-SSC Protocol No. 339). All participants gave written informed consent.

## Results

### Descriptive characteristics

**Fig. 1:**
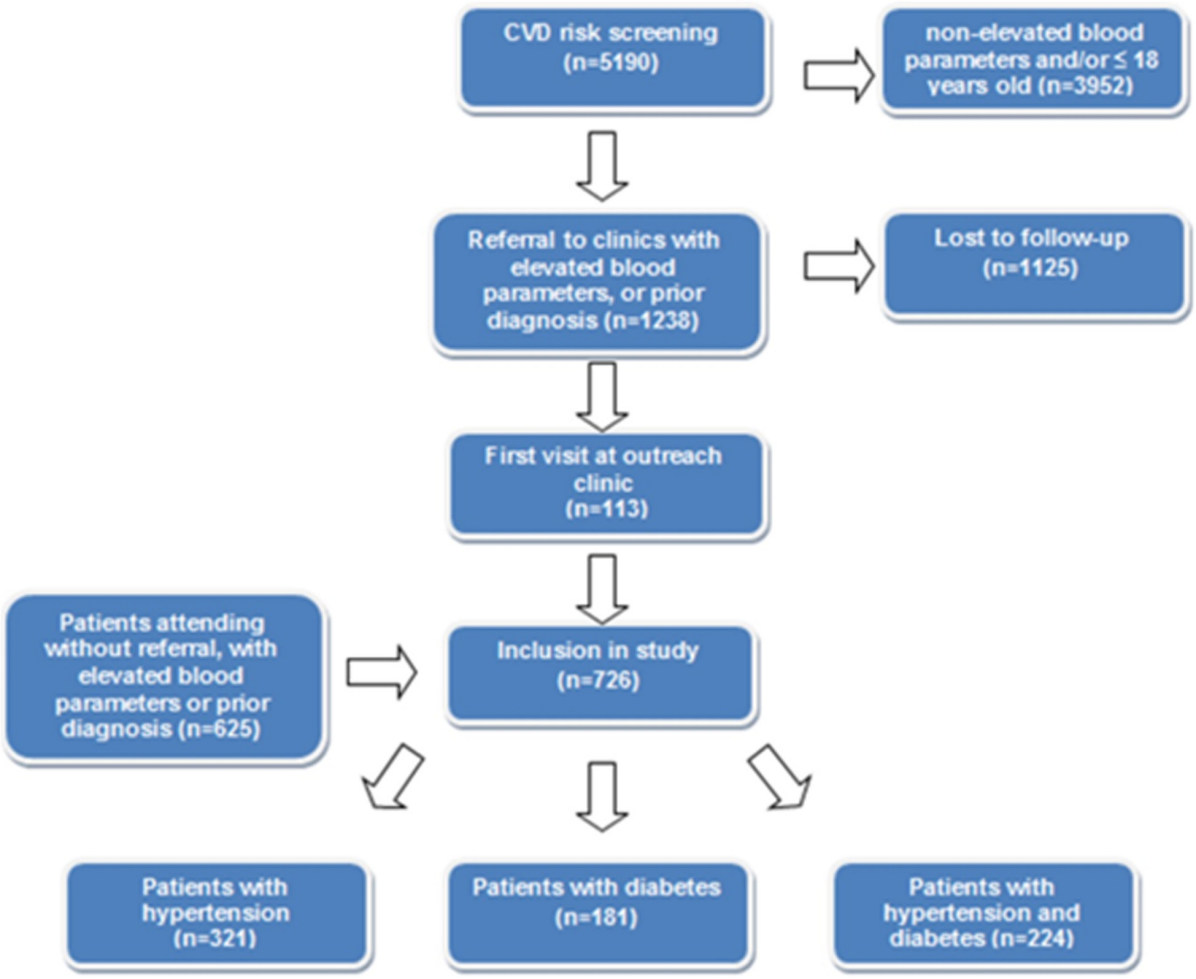
Flow chart of patients who met the inclusion criteria for participation in this study

Out of 5190 people screened in the CVD risk factor survey, 1238 were referred to the clinics of whom 113 (9.1 %) attended the clinics at least once. The rest of the participants (625 patients) attended the clinic without referral from the survey, resulting in a study population of 726 participants (see Fig. 1). At registration mean SBP among participants was 151.1 ± 28.9 mmHg, mean DBP was 89.2 ± 15.5 mmHg and mean RBG was 10.9 ± 7.2 mmol/L. The descriptive characteristics of the study population are presented in Tables 1 and 2. Up to 74 % (545 patients) of the participants were hypertensive. Among those with hypertension, mean SBP was 156.0 ± 28.9 mmHg while the mean DBP was 92.6 ± 15.9 mmHg. The overall prevalence of diabetes in this population was 55 %. Among patients with diabetes, 55 % (224 out of 405 patients) were hypertensive and among patients with hypertension 41 % (224 out of 545 patients) were diabetic.

**Table 1:** Demographic data in the study population at baseline

Demographics (*n* = 726)				
	All (*n* = 726)	HT only (*n* = 321)	DM only (*n* = 181)	HT&DM (*n* = 224)
Sex:				
Male	282 (38.8 %)	145 (45.2 %)	67 (37.0 %)	70 (31.3 %)
Female	444 (61.2 %)	176 (54.8 %)	114 (63.0 %)	154 (68.8 %)
Residence:				
Korogocho	122 (16.5 %)	43 (35.2 %)	35 (28.7 %)	44 (36.1 %)
Viwandani	269 (36.4 %)	150 (55.8 %)	58 (21.6 %)	61 (22.7 %)
Other	56 (7.6 %)	14 (25 %)	14 (25 %)	28 (50 %)
Missing	291 (39.4 %)	114 (35.5 %)	74 (40.9 %)	91 (40.6 %)
Referred:				
Yes	113 (15.3 %)	44 (13.7 %)	41 (22.7 %)	28 (12.5 %)
No	625 (84.7 %)	277 (86.3 %)	140 (77.3 %)	196 (87.5 %)

HT only, diagnosed with hypertension only; DM only, diagnosed with diabetes only; HT&DM, diagnosed with hypertension and diabetes

**Table 2:** Disease parameters in the study population at baseline

Prevalence and disease parameters				
Prevalence	Population	Male	Female	*P* value^*^ M. vs. F.
Hypertension	74.9 %	76.2 %	74 %	0.495
Diabetes	55.6 %	48.6 %	60.1 %	0.002
Disease parameters				
	All (*n* = 726)			
	Mean ± SD	Male ± SD	Female ± SD	P value^**^ (95 % CI) M. vs. F.
SBP (mmHg)	151.1 ± 28.9	153.4 ± 27.1	149.7 ± 29.9	0.109 (148.9-154.3)
DBP (mmHg)	89.2 ± 15.5	89.3 ± 15.2	89.2 ± 15.7	0.922 (88.0-90.4)
RBG (mmol/L)	10.9 ± 7.2	11.5 ± 7.3	10.6 ± 7.1	0.332 (10.1-11.8)
	Hypertension only (*n* = 321)			
	Mean ± SD	Male ± SD	Female ± SD	P value^**^(95 % CI) M. vs. F.
SBP (mmHg)	156.0 ± 28.9	159.7 ± 27.1	152.8 ± 30.0	0.036 (152.7-159.2)
DBP (mmHg)	92.6 ± 15.9	92.9 ± 15.1	92.4 ± 16.7	0.772 (90.8-94.4)
RBG (mmol/L)	6.3 ± 3.7	6.9 ± 3.3	6.0 ± 3.8	0.401 (5.4-7.2)
	Diabetes only (*n* = 181)			
	Mean ± SD	Male ± SD	Female ± SD	P value^**^ (95 % CI) M. vs. F.
SBP (mmHg)	139 ± 26.8	135.2 ± 21.9	141.0 ± 28.9	0.209 (134.7-143.2)
DBP (mmHg)	84.8 ± 14.6	81.8 ± 11.9	86.3 ± 15.6	0.066 (82.4-87.1)
RBG (mmol/L)	12.8 ± 8.6	14.7 ± 9.2	11.7 ± 8.0	0.111 (11.1-14.6)
	Hypertension and diabetes (n = 224)			
	Mean ± SD	Male ± SD	Female ± SD	P value^**^ (95 % CI) M. vs. F.
SBP (mmHg)	152.7 ± 27.8	154.0 ± 24.7	152.1 ± 29.2	0.646 (148.8-156.6)
DBP (mmHg)	87.3 ± 14.2	87.4 ± 15.2	87.3 ± 13.8	0.961 (85.3-89.3)
RBG (mmol/L)	11.8 ± 6.5	11.1 ± 5.8	12.2 ± 6.8	0.346 (10.7-12.9)

*SBP* systolic blood pressure, *DBP* diastolic blood pressure, *RBG* random blood glucose. HT only, diagnosed with hypertension only; DM only, diagnosed with diabetes only; HT&DM, diagnosed with hypertension and diabetes; SD, standard deviation; 95 % CI, 95 % confidence interval

^*^ *P* values were calculated using Chi-Square tests

^**^ *P* values were calculated using unpaired two-tailed *T*-test

Analysis by area of residence was performed since patients from different areas, and thus with different demographics, could participate. This showed that among patients with hypertension, mean SBP and DBP was highest in men from outside Korogocho and Viwandani (163.8 ± 29.7 mmHg and 96.6 ± 14.5 mmHg). Diabetic men from Viwandani had the highest mean RBGs (21.1 mmol/L).

For 2 different baseline data sets (containing participants who were in the program 6 and 12 months respectively) characteristics were also determined. In all patient groups females were overrepresented, ranging from 51.0 % of patients with hypertension who participated for at least 12 months to 63.0 % of patients with both diseases who participated for at least 12 months. Furthermore, in every patient group most people came from Viwandani (ranging from 43.6–71 % of participants), only for diabetics who were in the program for 12 months Korogocho was overrepresented (51.6 %).

### Patterns of clinic attendance and compliance

Findings on the patterns of clinic attendance for the entire study population over a 12-months period showed that up to 30 % of patients attended the clinic only once and 5 % of all patients attended only twice. Over a 12 month period, 3.4 % (25 patients) of all patients consistently attended the clinics every four weeks for a year. In this group, the incidence of having both hypertension and diabetes was 68 %. The rest of the patients had a more irregular pattern of clinic attendance. Conducting meaningful analyses beyond a 12 month period was not possible due to high dropout rates. The mean number of visits patients had in a year was 4.8 ± 3.9. Patients with hypertension only had 3.3 ± 3.3 visits in a year, those with diabetes only had 5.5 ± 3.7 and those with both diseases reached 6.5 ± 4.2 visits a year. When we divided patients in groups matching the number of appointments they had had during their participation in the program we found that patients who attended the clinics up to four times already had significant reductions in mean SBP (to 144 ± 28.5 mmHg *P* = .001) and in mean DBP (to 85.3 ± 14.7 mmHg, *P* = .001), regardless of compliance. Furthermore, we found that with increasing numbers of appointments disease parameters lowered, with patients who had 12 appointments reaching a mean SBP of 141.5 ± 25.5 mmHg (*P* = .003), and a mean DBP of 83.2 ± 13.7 mmHg (*P* < .001).

**Fig. 2:**
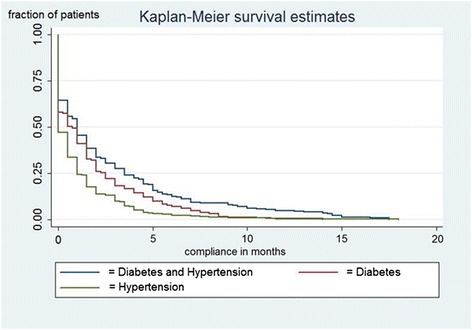
Survival analysis of compliance by the different diagnosed groups. The y-axis shows the fraction of patients by diagnosis, the x-axis shows the compliance over time in weeks. The blue line indicates patients with both diseases, the red line indicates patients with diabetes and the green line indicates patients with hypertension

In patients with hypertension, diabetes and both diseases, 75 % were non-compliant respectively after 4, 10 and 14 visits (HR 1.27, 95 % CI 1.17-1.39, *P* < 0.001). Figure 2 represents this visually. The differences in compliance between patients with hypertension and those with diabetes disappeared after approximately 8 months. After 15 months of treatment virtually everyone in every patient group had become non-compliant. Although men seemed less compliant than women, the median number of visits before patients became non-compliant was two for both sexes (HR 1.12, *P* = 0.151, 95 % CI 0.96-1.29). We investigated the effects of introducing a user fee as a possible distorting factor in the patterns of compliance and did not find any trend breaks, meaning that it did not have a significant effect on clinic attendance. Also, the average inflow of patients remained constant at 4 % after introduction of the fee.

### Changes in blood pressure and RBG

We found that within a subpopulation that committed to at least 12 clinic visits, the mean SBP decreased from 150.4 ± 28.5 mmHg to 141.5 ± 25.5 mmHg (*P* = .003) (Table 3). A decrease was also found for mean DBP in this group: from 89.3 ± 16.4 mmHg to 83.2 ± 13.7 mmHg (*P* = .001). For mean RBG no significant changes were observed for this group.

**Table 3:** Alteration of disease parameters after 6 and 12 months of treatment in the entire study population and stratified by diagnosis

	Mean ± SD baseline 6 months	Mean ± SD 6 months (Δ)	*P* value* (95 % CI)	Mean ± SD baseline 12 months	Mean ± SD 12 months (Δ)	*P* value* (95 % CI)
All patients	(*n* = 237)			(*n* = 154)		
SBP (mmHg) ± SD	148.5 ± 28.3	139.4 ± 25.4 (Δ -9.1)	<0.001 (141.5-146.4)	150.4 ± 30.3	141.5 ± 25.5 (Δ -8.9)	0.003 (142.7-149.1)
DBP (mmHg) ± SD	88.0 ± 15.4	82.9 ± 15.3 (Δ -5.1)	0.002 (84.0-86.8)	89.3 ± 16.4	83.2 ± 13.7 (Δ -6.1)	<0.001 (84.5-87.9)
RBG (mmol/L) ± SD	11.3 ± 6.7	11.3 ± 8.8 (Δ -0.0)	0.5308 (10.5-12.1)	10.7 ± 6.1	11.8 ± 6.8 (Δ +1.1)	0.909 (10.4-12.1)
Hypertension only	(*n* = 65)			(*n* = 33)		
SBP (mmHg) ± SD	158.2 ± 26.0	136.2 ± 25.9 (Δ -22.0)	<0.001 (142.5-152.2)	160.7 ± 32.1	139.3 ± 27.4 (Δ -21.4)	0.003 (142.5-158.1)
DBP (mmHg) ± SD	94.8 ± 15.1	82.9 ± 15.4 (Δ -11.9)	<0.001 (86.1-91.7)	96.9 ± 19.6	84.1 ± 13.9 (Δ -12.8)	0.002 (86.2-95.1)
RBG (mmol/L) ± SD	6.5 ± 3.3	7.1 ± 4.5 (Δ +0.6)	0.738 (5.7-7.8)	8.4 ± 5.2	10.5 ± 6.9 (Δ +2.1)	0.789 (6.8-11.9)
Diabetes only	(*n* = 69)			(*n* = 40)		
SBP (mmHg) ± SD	139.9 ± 30.6	136.0 ± 20.6 (Δ -3.9)	0.192 (133.6-142.4)	140.1 ± 33.7	132.5 ± 23.9 (Δ -7.6)	0.132 (129.6-142.9)
DBP (mmHg) ± SD	85.5 ± 15.1	82.9 ± 16.6 (Δ -2.6)	0.171 (81.5-86.9)	87.8 ± 15.7	82.5 ± 13.1 (Δ -5.3)	0.058 (81-7-88.4)
RBG (mmol/L) ± SD	12.6 ± 7.7	11.5 ± 6.4 (Δ -1.1)	0.203 (10.7-13.3)	10.2 ± 6.5	11.5 ± 7.3 (Δ +1.3)	0.774 (9.2-12.6)
Hypertension & diabetes	(*n* = 100)			(*n* = 80)		
SBP (mmHg) ± SD	147.7 ± 26.7	143.9 ± 27.8 (Δ -3.8)	0.161 (142.0-149.6)	152.3 ± 26.7	146.7 ± 25.1 (Δ -5.6)	0.092 (145.3-153.6)
DBP (mmHg) ± SD	85.2 ± 14.6	83.4 ± 14.2 (Δ -1.8)	0.180 (82.3-86.3)	87.3 ± 14.6	83.1 ± 12.7 (Δ -4.2)	0.028 (83.0-87.4)
RBG (mmol/L) ± SD	11.9 ± 6.2	12.1 ± 10.6 (Δ +0.2)	0.558 (10.7-13.3)	11.6 ± 6.1	12.4 ± 6.8 (Δ +0.8)	0.750 (10.9-13.1)

*SBP* systolic blood pressure, *DBP* diastolic blood pressure, *RBG* random blood glucose *SD* standard deviation, Δ difference between baseline means and means after 6 or 12 months, 95 % *CI* 95 % confidence interval

*P value calculated with paired two-tailed *T*-test after 6 and 12 months in comparison with the corresponding baseline value

Among patients with only hypertension we found significant reductions of the systolic (from 160.7 ± 32.1 mmHg to 139.3 ± 27.4 mmHg, *P* = .003) and diastolic blood pressure (from 96.9 ± 19.6 mmHg to 84.1 ± 13.9 mmHg, *P* = .002) after one year of clinic attendance. For patients with diabetes and those with both diseases there were no significant reductions in systolic blood pressure or RBG after 12 months of clinic attendance. DBP lowered in patients with both diseases from 87.3 ± 14.6 mmHg to 83.1 ± 12.7 mmHg (*P =* 0.028) (Table 3). When we conducted the same analysis for patients who were in the program for 6 months we found a decrease in SBP and DBP for patients with hypertension (Table 3). For the other patient groups, no significant alterations were found after 6 months.

This led to hypertension control rates for SBP at baseline, after 6 months and after 12 months of treatment of 31 %, 63 % and 32 % respectively. For DBP control rates lowered from 50 % at baseline to 46 % after 6 months and 38 % after 12 months of treatment. In this study, the treatment effects for men and women in all patient groups were similar.

When stratifying for area of residence it appeared that the mean SBP and DBP in Korogocho and Viwandani significantly decreased after one year. RBG was raised in every location.

## Discussion

The aim of this study was to investigate the effect of primary health care clinics, set up to treat hypertension and diabetes in two Nairobi slums, on clinic attendance patterns and clinical parameters among the attendees. Key findings are that while there was an overall reduction in mean blood pressure for patients that were still in the project after 12 months, compliance with care was very poor.

In recent years it has become clear that prevention programs for CVD in low- and middle income countries are cost-effective [20] and scalable when targeting important risk factors [24, 25]. Several succesful interventions have been implemented in low-income settings to tackle the increasing burden of non-communicable diseases, specifically CVD [26, 27]. However, a study which evaluated the attendance of an intervention similar to the one in this study, carried out in Dar es Salaam in 2008 suggests low use of health care services and poor treatment compliance after 12 months [28]. In this case, treatment costs and lack of symptoms were reasons reported for not attending care.

In our study population it was remarkable that although people were referred to the clinics from a mass screening campaign, only a small fraction of them attended the clinic (113 out of 1238), and the majority of patients attended on their own accord without referral. This shows that there are still challenges to overcome in order to successfully implement a screen-and-treat intervention approach in low-resource settings. It is not clear why such a small fraction of referred patients showed up at the clinic even though they were aware that the cost of care was initially free. A separate study by APHRC in Korogocho slum [29] which involved door-to-door screening for hypertension found that the reasons given for non-attendance to clinics after referral include lack of time or money, forgetfulness, and lack of understanding of health benefits of attendance (APHRC, unpublished data). The large proportion of un-referred patients attending the clinics points to a large unmet need for care among patients already diagnosed and perhaps more aware of the benefits of treatment. This could also point to barriers to affordability of treatment. Treatment costs for hypertension or diabetes outside the slums could be as high as 10 to 20 times the user fee that was introduced at both clinics.

From the patterns of attendance we observed that only a small percentage of participants in this study visited the clinics consistently over the 12-month follow up period. These patterns also show high dropout rates, in particular after the first visit (27 %) of which the vast majority were patients with hypertension (67 %). Survival analysis demonstrates that patients with hypertension are the least compliant. Possible reasons for high dropout rates and low compliance of patients with hypertension might be the lack of symptoms in hypertension, low awareness and a low priority for treatment [28]. These findings are consistent with earlier studies, which state that drop-out rates in low-income settings are often high, in particular when no symptoms are reported or when treatment costs are substantial [28, 30]. However, the introduction of the user fee in this study did not affect clinic attendance. This finding is partly attributable to the fact that the majority of patients had already dropped out and no new patients had been recruited in the six months prior to the introduction of the fees. Another possible contributor to the high dropout rates might have been that when blood pressure or blood glucose was controlled or improving, people considered themselves cured and stopped attending the clinics. In our study population we found that patients who attended the clinics for longer periods of time had higher mean blood pressures at baseline, implying that patients with lower blood pressures might drop out early, and possibly, that patients who have blood pressures more resistant to treatment stay in the program longer.

We observed that in the entire study population, although dropout rates were very high, the systolic and diastolic blood pressure had significantly lowered after 12 months. However, this population comprised patients with both regular and irregular attendance patterns. Patients who had had more visits showed more significant decreases, making it plausible that the impact on disease parameters increases when patients attend the clinics more often. A possible explanation for this finding is that people who attend more regularly are more likely to also take their medication more regularly and are likely to be more stimulated to commit to lifestyle changes.

The program seemed to have had positive effects for people with hypertension: after one year of clinic attendance there were significant reductions in mean SBP and DBP. One possible explanation for this is that when patients receive treatment, the rate of control of hypertension can rise to nearly 40 % [31]. However, when looking at control rates for hypertension, it seemed that although we found a steep increase for the control rate of SBP after 6 months of treatment, control rates reached baseline values again after 12 months of treatment. The fact that these effects disappeared later, emphasizes the difficulties of long-term disease management in these settings.

A quite consistent finding in this study is the gradual rise of RBG over time. This might be due to low compliance rates in the study population and possibly to low compliance to medication intake, which was not measured in this study. It also reflects the fact that such diseases may require intensive disease management, which could be quite challenging in low-resource settings.

There were some limitations to this study. Given the important reduction in the number of patients returning for follow-up visits, we had to work with small populations in certain analyses. This drastically limits the external validity of our findings on the effects on blood parameters. Also, we used RBG as a disease parameter which is not an accurate measure because of high inter- and intrapersonal fluctuations. HbA1c tests, which are more reliable in determining glycemic control over time, were available, but not free and the majority of patients decided not to get tested. Therefore, analyzing HbA1c was not possible in the study population. RBG is used as a starting point for referral because its low cost and quick determination of blood glucose values make it an easy-to-use method for glucose measurement in the field.

## Conclusions

This study demonstrates that establishing a preventative care and treatment system in low resource settings for CVD is challenging due to high dropout rates and non-compliance. To address this problem, it is important to raise awareness of treatment compliance in slums since non-communicable diseases such as CVD often need lifelong treatment. The study also demonstrated that a screen-and-treat based approach for referral and treatment was not very effective in this poor population. Overall, there is urgent need for more research in innovative approaches to improve patient compliance in these challenging settings.

## Abbreviations

APHRC: African population and health research center
DBP: Diastolic blood pressure
CVD: Cardiovascular disease
LMIC: Low- and middle income countries
SBP: Systolic blood pressure
RBG: Random blood glucose
